# Low hemoglobin is associated with poor functional outcome after non-traumatic, supratentorial intracerebral hemorrhage

**DOI:** 10.1186/cc8961

**Published:** 2010-04-14

**Authors:** Jennifer Diedler, Marek Sykora, Philipp Hahn, Kristin Heerlein, Marion N Schölzke, Lars Kellert, Julian Bösel, Sven Poli, Thorsten Steiner

**Affiliations:** 1 Department of Neurology, University of Heidelberg, Im Neuenheimer Feld 400, 69120 Heidelberg, Germany; 2 Department of Neurology, Comenius University, Mickiewiczova 13, 813 69 Bratislava, Slovakia

## Abstract

**Introduction:**

The impact of anemia on functional outcome and mortality in patients suffering from non-traumatic intracerebral hemorrhage (ICH) has not been investigated. Here, we assessed the relationship between hemoglobin (HB) levels and clinical outcome after ICH.

**Methods:**

One hundred and ninety six patients suffering from supratentorial, non-traumatic ICH were extracted from our local stroke database (June 2004 to June 2006). Clinical and radiologic computed tomography data, HB levels on admission, mean HB values and nadir during hospital stay were recorded. Outcome was assessed at discharge and 3 months using the modified Rankin score (mRS).

**Results:**

Forty six (23.5%) patients achieved a favorable functional outcome (mRS ≤ 3) and 150 (76.5%) had poor outcome (mRS 4 - 6) at discharge. Patients with poor functional outcome had a lower mean HB (12.3 versus 13.7 g/dl, *P *< 0.001) and nadir HB (11.5 versus 13.0 g/dl, *P *< 0.001). Ten patients (5.1%) received red blood cell (RBC) transfusions. In a multivariate logistic regression model, the mean HB was an independent predictor for poor functional outcome at three months (odds ratio (OR) 0.73, 95% confidence interval (CI) 0.58-0.92, *P *= 0.007), along with National Institute of Health Stroke Scale (NIHSS) at admission (OR 1.17, 95% CI 1.11 - 1.24, *P *< 0.001), and age (OR 1.08, 95% CI 1.04 - 1.12, *P *< 0.001).

**Conclusions:**

We report an association between low HB and poor outcome in patients with non-traumatic, supratentorial ICH. While a causal relationship could not be proven, previous experimental studies and studies in brain injured patients provide evidence for detrimental effects of anemia on brain metabolism. However, the potential risk of anemia must be balanced against the risk of harm from red blood cell infusion.

## Introduction

Intracerebral hemorrhage (ICH) accounts for approximately 10 to 15% of acute strokes and is still associated with a mortality up to 30 to 50% [1]. ICH volume, neurological status on admission, age above 80 years and the presence of intraventricular blood were found to be strong predictors of 30-day mortality [2]. Around 50% of the patients require mechanical ventilation [3] and most are admitted to an ICU [4]. A study including medical and surgical ICU patients found a high incidence of anemia in critically ill patients and the nadir hemoglobin (HB) level of less than 9 g/dl as a predictor of increased mortality and length of hospital stay [5]. At the same time, the number of red blood cell (RBC) transfusions a patient received was independently associated with increased mortality. The current literature supports the idea that many critically ill patients tolerate HB levels as low as 7 g/dl and that a liberal transfusion strategy may in fact lead to worse clinical outcome [5,6]. However, it remains unclear whether a restrictive transfusion threshold is also suited for neurocritical care patients. Studies including patients with subarachnoid hemorrhage (SAH) [7-9] or traumatic brain injury (TBI) [10-12] provide evidence that low HB is associated with poor functional outcome. A recent study in SAH patients reports that higher HB levels (11.7 ± 1.5 g/dl vs. 10.9 ± 1.2 g/dl) were related with better outcome at discharge and at three months [7]. The effects of anemia in patients suffering from supratentorial non-traumatic ICH have not yet been investigated. In the current study, we assessed the impact of anemia on functional outcome and mortality after ICH.

## Materials and methods

### Patients

We retrieved all patients suffering from supratentorial ICH that were admitted to our stroke unit or neurological ICU between June 2004 and June 2006 from our local stroke database (n = 247). Complete datasets including computed tomography (CT) data, baseline National Institutes of Health Stroke Scale (NIHSS), modified Rankin Scale (mRS) at discharge and laboratory tests were available for 196 patients. ICH was diagnosed by CT. Hematoma volume was calculated from the first CT scan using the a × b × c × 0.5 method [13]. Stroke severity on admission was assessed using the NIHSS. Functional outcome at discharge was assessed by the attending physician using the mRS. Functional outcome at three months was assessed by a standardized telephone interview using the mRS or by assessing the final reports after end of rehabilitation. Outcome scores were dichotomized into favorable (mRS ≤ 3) and poor functional outcome (mRS 4-6). The study was approved by the local ethics committee (S-406/2009). The data was collected in an anonymized database, therefore the need for informed consent was waived.

### HB levels and transfusions

All HB measurements during hospital stay were extracted from the laboratory database. Routinely, ICU patients have daily laboratory controls, stroke unit patients every other day. In the mean, 7.5 HB values were available for each patient (SD ± 8.0). For every patient, HB on admission and the nadir (lowest value during hospital stay) and mean HB levels (calculated from all available values) were recorded for further analysis. The records of all patients with a nadir of 10 g/dl or less were screened for RBC transfusions and the reason for transfusion. At the moment there is no predefined institutional standard for RBC transfusion in ICH patients. Anemia was defined as a HB level of less than 12.1 g/l for women and less than 13.1 g/l for men [14].

### Statistical analysis

Distribution of the data was tested using the one-sample Kolmogorov-Smirnov test. Comparisons between groups of patients were made using the chi-squared, Student's *t *test or the Mann-Whitney U test where appropriate. Mean HB values in the different outcome categories were compared by using a one-way analysis of variance (ANOVA), *post-hoc *analyses were performed applying Bonferroni adjustment. Homogeneity of variance was tested using the Levene's homogeneity-of-variance test when applying the Student's *t *test and ANOVA. Logistic regression was used to create a model to predict poor outcome (mRS 4 to 6) at discharge and at three months and in-hospital mortality. The variables age, hemorrhage volume, NIHSS at admission, the presence of intraventricular blood, ICU stay, the need for mechanical ventilation, RBC transfusion, the mean HB level during hospital stay and admission HB were entered into the multivariate model. Because of co-linearity of the included variables, a forward stepwise regression analysis was employed to select which of the variables should be included in a regression model to predict the dependent variable. The procedure applied the variables one by one using a criterion such that the F-statistic for the variable to be added must exceed the level of 0.05. After a variable has been added to the model, the procedure analyzes all included variables and deletes any variable that fails to produce an F-value less than 0.1. After necessary deletions are accomplished, further variable can be added to the model. The procedure terminates when no variable outside the model exceeds the necessary threshold and every variable included is significant. A Cox proportional model was used in order to analyse the risk to develop anemia during hospital stay for both outcome groups. Values of *P *< 0.05 were considered statistically significant in all tests. Statistical analyses were performed using the SPSS software package (SPSS 16.0, SPSS Inc., Chicago, Illinois, USA).

## Results

The mean age was 67.1 years (range 29 to 96, standard deviation (SD) 13.5). The median hemorrhage volume was 26.9 ml (range 0.4 to 383, interquartile range (IQR) 60.8) and 78 patients (39%) had intraventricular hemorrhage extension. The most frequent etiologies were hypertension (43%) and ICH due to oral anticoagulants (20%); other etiologies included amyloid angiopathy (16%) and arteriovenous malformations or cavernous angioma (6%). For 7.1% of patients the etiology remained undetermined at the time of discharge.

The mean admission HB was 13.7 g/dl (range 7.4 to 18.4, SD 1.9), the mean HB during hospital stay was 12.6 g/dl (range 6.3 to 18.4, SD 2.1) and the mean nadir HB was 11.9 g/dl (range 6.3 to 18.4, SD 2.3). Only 10 patients (5%) received RBC transfusions during hospital stay. Mean admission HB of transfused patients was 12.4 g/dl (7.6 to 16.3, SD 2.7) and mean nadir HB triggering transfusion was 7.9 g/dl (6.3 to 10, SD 1.2). Reasons for transfusion were surgery (n = 5), erosive gastritis (n = 1), anemia due to chronic nephropathy (n = 1), coronary artery disease (n = 1) and undetermined (n = 2). None of the included patients encountered major bleedings with the need for mass transfusion.

Table 1 shows the univariate analysis of outcome at discharge. Fourty-six (24%) patients achieved a favorable functional outcome (mRS ≤ 3) while 150 (77%) had a poor functional outcome (mRS 4 to 6). Patients with poor functional outcome were significantly older (62.5 vs. 68.6 years, *P *= 0.007) had larger hemorrhages (8.6 vs. 30 ml, *P *< 0.001), and a lower mean HB (13.7 vs. 12.3 g/dl, *P *< 0.001) and nadir HB (13.0 vs. 11.5 *P *< 0.001) during hospital stay. In addition, significantly more patients with poor outcome had intraventricular hemorrhage extension (11% vs. 48%, *P *< 0.001), developed anemia during hospital stay (22% vs. 56%, *P *< 0.001), needed ICU treatment (15 vs. 51%, *P *< 0.001), and mechanical ventilation (4% vs. 37%, *P *< 0.001). There was no significant difference in the median duration of hospital stay (seven vs. six days, *P *= 0.32) or the number of transfused patients between both groups (2% vs.6%, *P *= 0.302).

**Table 1:** Univariate analysis of functional outcome at discharge

Variable	mRS 0 to 3 (n = 46)	mRS 4 to 6 (n = 150)	*P*
Age (years) (mean, SD)	62.5 (13.4)	68.6 (13.1)	0.007^a^
Sex (male, n, %)	29 (63.0)	101 (67.3)	0.136^c^
Hemorrhage volume (ml) (median, IQR)	8.6 (24.0)	30 (66.4)	<0.001^b^
Intraventricular hemorrhage extension (n, %)	5 (10.9)	72 (48.0)	<0.001^c^
NIHSS on admission (median, IQR)	4 (3)	16 (23)	<0.001^b^
Admission HB (g/dl) (mean, SD)	14.2 (1.6)	13.6 (2.0)	0.052^a^
Mean HB (g/dl) (mean, SD)	13.7 (1.8)	12.3 (2.0)	<0.001^a^
Nadir HB (g/dl) (mean, SD)	13.0 (1.9)	11.5 (2.3)	<0.001^a^
Anemia during hospital stay (n, %)	10 (21.7)	84 (56.0)	<0.001^c^
HB measurements (n) (median, IQR)	4 (4.3)	5 (8.5)	0.22 ^b^
Hospital stay (days) (median, IQR)	7 (8)	6 (8)	0.32^b^
ICU stay (n, %)	7 (15.2)	76 (50.7)	<0.001^c^
Mechanical ventilation (n, %)	2 (4.3)	55 (36.9)	<0.001^c^
RBC transfusion (n, %)	1 (2.2)	9 (6.0)	0.302^c^

^a ^Student *t*-test

^b ^Mann-Whitney-U test

^c ^Chi squared test

HB, hemoglobin; IQR, interquartile range; mRS, modified Rankin Scale; NIHSS, National Institutes of Health Stroke Scale; RBC, red blood cell; SD, standard deviation.

**Figure 1:**
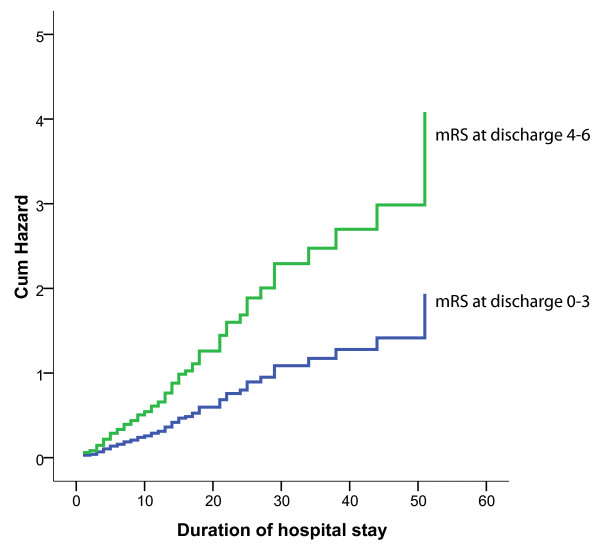
**Cumulative hazard to develop anaemia during hospital stay for both outcome groups separately**. During their hospital stay, patients with poor functional outcome (modified Rankin score (mRS) 4 to 6) had a higher risk to develop anemia compared with those with favorable outcome at discharge.

Figure 1 shows the risk to develop anemia during hospital stay for patients with favorable (mRS 0 to 3) versus poor (mRS 4 to 6) functional outcomes. Poor functional outcome at discharge was associated with a higher risk of developing anemia throughout hospital stay (*P *= 0.029).

**Figure 2:**
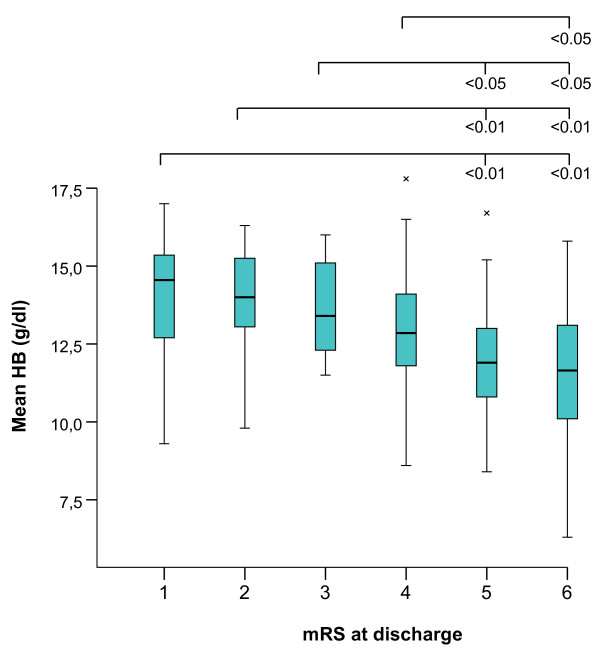
**Mean hemoglobin values and outcome at discharge**. Mean hemoglobin (HB) levels were significantly different between all groups (*F *(5,172) = 7.71, *P *< 0.001). Significant differences between group means are indicated by the bars (*post-hoc *analysis using Bonferroni adjustment for multiple comparisons). mRS, modified Rankin score.

Figure 2 shows patients stratified by mRS category (1 to 6), excluding the patients that did not survive the first day in hospital (n = 17). Mean HB levels were significantly different between groups (14.0, 14.0, 13.4, 13.0, 11.9, and 11.2 mg/dl for mRS 1 to 6 respectively, *F *(5,172) = 7.71, *P *< 0.001). *Post-hoc *analysis to compare the different outcome groups separately was performed using Bonferroni adjustment for multiple comparisons; *P *values are indicated in Figure 2.

We performed a stepwise logistic regression model to predict unfavorable outcome (mRS 4 to 6) at discharge, including age, hemorrhage volume, baseline NIHSS, the presence of intraventricular hemorrhage extension, ICU stay, the need for mechanical ventilation, RBC transfusion and the mean HB level during hospital stay. In the final model, only NIHSS on admission (odds ratio (OR) 1.30, 95% confidence interval (CI) 1.17 to 1.45, *P *< 0.001), presence of intraventricular hemorrhage extension (OR 4.50, 95% CI 1.23 to 15.83, *P *= 0.019), age (per year, OR 1.04, 95% CI 1.01 to 1.08, *P *= 0.021) and the mean HB (per mg/dl, OR 0.76, 95% CI 0.59 to 0.97, *P *= 0.027) remained independent predictors for poor functional outcome at discharge (Table 2).

**Table 2:** Final stepwise logistic regression model to predict poor outcome (mRS 4 to 6) at discharge. Included variables: age, hemorrhage volume, NIHSS at admission, the presence of intraventricular blood, ICU stay, the need for mechanical ventilation, RBC transfusion and the mean HB level during hospital stay

Variable	Coefficient	*P*	OR (95% CI)
NIHSS on admission (per point)	0.261	<0.001	1.30 (1.17-1.45)
Presence of intraventricular hemorrhage extension	1.503	0.019	4.50 (1.23-15.83)
Age (per year)	0.043	0.021	1.04 (1.01-1.08)
Mean HB (per mg/dl)	-0.281	0.027	0.76 (0.59-0.97)

CI, confidence interval; HB, hemoglobin; mRS, modified Rankin Scale; NIHSS, National Institutes of Health Stroke Scale; OR, odds ratio; RBC, red blood cell.

In-hospital mortality was 22% (43 patients). In univariate analysis, hemorrhage volume, intraventricular hemorrhage extension, admission status, duration of hospital stay, the need for mechanical ventilation and ICU stay, and admission HB levels were associated with in-hospital mortality (Table 3). In the multivariable logistic regression model, NIHSS on admission (OR 1.17, 95% CI 1.10 to 1.24, *P *< 0.001), hemorrhage volume (per ml, OR 1.02, 95% CI 1.01 to 1.03, *P *= 0.003) and age (per year, OR 1.05, 95%CI 1.02 to 1.09, *P *= 0.006) remained independent predictors for in-hospital mortality (Table 4).

**Table 3:** Univariate analysis of factors associated with in-hospital mortality

Variable	mRS 0 to 5 (n = 153)	mRS 6 (n = 43)	*P*
Age (years) (mean, SD)	66.1 (13.8)	71.1 (11.1)	0.028^a^
Sex (male, n, %)	100 (65.4)	30 (69.8)	0.589^c^
Hemorrhage volume (ml) (median, IQR)	17.1 (36.5)	88 (108.9)	<0.001^b^
Intraventricular hemorrhage extension (n, %)	49 (32.9)	28 (65.1)	<0.001^c^
NIHSS on admission (median, IQR)	10 (12)	34 (10)	<0.001^b^
Admission HB (g/dl) (mean, SD)	13.9 (1.8)	13.2 (2.3)	0.040^a^
Mean HB (g/dl) (mean, SD)	12.8 (2.0)	12.2 (2.4)	0.150^a^
Nadir HB (g/dl) (mean, SD)	11.9 (2.2)	11.7 (2.7)	0.516^a^
Anemia during hospital stay (n, %)	71 (46.4)	23 (53.5)	0.411^c^
Hospital stay(days) (median, IQR)	7 (9)	2 (43)	<0.001^b^
ICU stay (n, %)	53 (34.6)	30 (69.8)	<0.001^c^
Mechanical ventilation (n, %)	33 (21.6)	24 (57.1)	<0.001^c^
RBC transfusion (n, %)	7 (4.6)	3 (7)	0.527^c^

^a ^Student *t*-test

^b ^Mann-Whitney-U test

^c ^Chi squared test

HB, hemoglobin; IQR, interquartile range; mRS, modified Rankin Scale; NIHSS, National Institutes of Health Stroke Scale; RBC, red blood cell; SD, standard deviation.

**Table 4:** Final stepwise logistic regression model to predict in-hospital mortality. Included variables: age, hemorrhage volume, NIHSS at admission, the presence of intraventricular blood, ICU stay, the need for mechanical ventilation, RBC transfusion and the admission HB

Variable	Coefficient	*P*	OR (95% CI)
NIHSS on admission (per point)	0.109	<0.001	1.17 (1.11-1.24)
Hemorrhage volume (per ml)	0.015	0.003	1.02 (1.01-1.03)
Age (per year)	0.052	0.006	1.05 (1.02-1.09)

CI, confidence interval; HB, hemoglobin; NIHSS, National Institutes of Health Stroke Scale; OR, odds ratio; RBC, red blood cell.

Outcome at three months was available for 176 (90%) patients, 20 patients were lost to follow up. Stepwise logistic regression revealed NIHSS on admission (OR 1.17, 95% CI 1.11 to 1.24, *P *< 0.001), age (per year, OR 1.08, 95% CI 1.04 to 1.12, *P *< 0.001) and the mean HB (per mg/dl, OR 0.73, 95% CI 0.58 to 0.92, *P *= 0.007) as independent predictors of outcome at three months (Table 5).

**Table 5:** Final stepwise logistic regression model to predict poor outcome (mRS 4 to 6) at three months. Included variables: age, hemorrhage volume, NIHSS at admission, the presence of intraventricular blood, ICU stay, the need for mechanical ventilation, RBC transfusion and the mean HB level during hospital stay)

Variable	Coefficient	*P*	OR (95% CI)
NIHSS on admission (per point)	0.157	<0.001	1.17 (1.11-1.24)
Age (per year)	0.076	<0.001	1.08 (1.04-1.12)
Mean HB (per mg/dl)	-0.319	0.007	0.73 (0.58-0.92)

CI, confidence interval; HB, hemoglobin; NIHSS, National Institutes of Health Stroke Scale; OR, odds ratio; RBC, red blood cell.

## Discussion

In the current study we found lower HB concentrations to be an independent predictor of poor functional outcome in patients suffering from supratentorial, non-traumatic ICH. Anemia during hospital stay was more likely to occur in patients with poor rather than with favorable functional outcomes.

The design of the current study does not allow for the determination of whether the relation between low HB levels and functional outcome is causative. Based on the current data, it cannot be excluded that anemia simply represents a marker for severity of illness, rather than causing additional brain injury. Critically ill patients usually receive more intravenous fluids, have more blood drawn for laboratory tests, and more frequently undergo invasive procedures, all factors leading to lower HB concentrations. However, we found lower HB concentrations to be associated with poor outcome, independent of stroke severity, which is the most powerful predictor of outcome of acute ICH.

The absolute difference of mean HB levels between both outcome groups was 1.4 mg/dl, corresponding to a reduction of blood oxygen content of around 10% ((1.39 × HB concentration - O_2 _Sat/100) + (0.003 × PaO_2_)). A reduction of mean HB levels from 14 to 11.9 mg/dl as found for patients with mRS 1 versus 6 respectively makes a difference in blood oxygen content of 20%, roughly assuming similar partial pressure of arterial oxygen (PaO_2_) levels. Previous studies in SAH patients report a comparable magnitude of absolute differences in HB levels between outcome groups [7,8]. Moreover, there is a large body of literature including patients with TBI [10-12], SAH [7-9], or ischemic stroke [15-17] suggesting that anemia or even relative anemia may not be tolerated in the setting of acute brain injury. In patients with acute brain injury, physiological compensatory mechanisms such as an increase in cerebral blood flow [18,19] may fail, rendering them more vulnerable to fluctuations in blood oxygen content. Possible mechanisms include impairment of cerebrovascular autoregulation and metabolic disturbances of the injured brain [20]. Although some studies have reported intact autoregulation in the perihematomal region in the acute and subacute phase of ICH [21,22], it has recently been demonstrated that global cerebral autoregulation can be impaired in ICH patients. Additionally, loss of cerebrovascular pressure reactivity was associated with poor outcome [23]. Failure of autoregulation may impede a compensatory increment in cerebral blood flow as response to anemia and thus render ICH patients more vulnerable to a decrease in blood oxygen content.

In addition, animal studies provide evidence that anemic hypoxia may exacerbate primary neurological injury [24,25]. Although the concept of an ischemic penumbra in hemorrhagic stroke has increasingly been challenged [26,27], new hypotheses claim the presence of a metabolic penumbra [28,29]. Recent studies do not point towards a general lack of oxygen in the perihematomal region, but rather to a changed metabolism [29] with low rates of oxygen use [26,30]. However, the exact timing of metabolic and inflammatory processes in the perihematomal zone remains to be elucidated. Moreover, it is conceivable that the metabolic and oxygen demand may change during the course of the disease. So far, there are no studies in humans suffering from acute ICH investigating the effect of low HB levels on the perihematomal zone. Only animal studies in dogs that were exposed to chronic anemia before the induction of experimental ICH provide evidence for an altered brain metabolism in anemic animals [31,32]. Although chronic anemic hypoxia promoted a decrease in metabolic demands and an increase in cerebral blood flow, these adaptive responses deteriorated at induction of ICH [31]. In the acute phase of ICH, anemia was associated with an increased critical threshold of brain oxygenation and progressive deterioration of cerebral hemodynamics.

Although we found an independent association between low HB and worse functional outcomes, HB levels during hospital stay were not predictive of in-hospital mortality. Only hemorrhage volume, age and admission status remained independent predictors for in-hospital mortality in the multivariate model. This may be explained by the overwhelming influence of hemorrhage volume on mortality and the fact that 40% of patients who died, died on the first day of hospital stay. Of interest, admission HB levels differed significantly between both groups in an univariate analysis. This finding is in line with a recent study including almost 700 patients with non-traumatic ICH investigating the role of anemia on admission (day 1) on the clinical course of acute ICH [33]. Although patients with anemia on admission (25.8% of patients) were at higher risk of death at 30 days in univariate analysis, this effect did not persist in a multivariate model including hemorrhage volume. Interestingly, the authors report that the presence of anemia on admission was associated with larger ICH volume and thereby hypothesize that the presence of anemia may contribute to hemorrhage growth. Another explanation may be that admission HB levels rather are a marker for poor physiological status on admission. Unfortunately, scores for physiologic illness such as the acute physiology and chronic health evaluation (APACHE) II score were not available due to the retrospective design of our study.

The main limitations of the current study include the small number of transfused patients and the retrospective, observational design that does not shed light on the underlying metabolic processes. However, while the latter was beyond the scope of the current study, further studies invasively assessing the parenchymal metabolic effects of anemia and RBC transfusion in ICH patients seem justified. In the current study, only 10 (5.1%) patients received RBC transfusions during their hospital stay. RBC transfusion was included as a variable in multivariable models; however, the number of transfused patients was too low to provide solid data on the effect of RBC transfusion on outcome parameters. In order to exclude the possibility that poor outcomes were primarily related to transfusion, rather than anemia, we repeated our analysis after excluding the 10 transfused patients from the multivariate models. In this repeat analysis, mean HB did not remain an independent predictor for poor outcome at discharge but stayed an independent predictor in the model for outcome at three months [see Additional file 1]. However, evidence exists from previous studies including patients with TBI [10,12] or SAH [34,35] that despite some putative beneficial physiological effects, RBC transfusion was associated with additional risks and poorer outcome. In addition, Zygun and colleagues recently have assessed the effect of RBC transfusion on cerebral oxygenation and metabolism in TBI patients [36]. They report that transfusion of RBC resulted in improved brain tissue oxygenation, but without noticeable effect on cerebral metabolism as measured by lactate-pyuvate ratio. There is currently no data available for ICH patients.

## Conclusions

In summary, in the current study we found an association between low HB and poor functional outcome in patients with non-traumatic ICH, as was previously reported for patients with SAH, TBI and ischemic stroke. Although none of the studies in brain-injured patients has so far proven a causative relation between anemia and poor outcome, physiological and observational studies provide evidence for possible detrimental effects of anemia on brain metabolism. However, the potential risk of anemia must be balanced against the potential risk of harm from allogenic RBC infusion. Further trials are needed to investigate the local metabolic effects of anemia and RBC transfusion in ICH patients.

## Key messages

• Poor functional outcome at discharge and at 90 days was associated with lower mean HB levels during hospital stay in patients with non-traumatic, supratentorial ICH.

• Based on the currently available data it could not be elucidated if the presence of anemia promotes further brain injury or if it represents a marker of severe illness.

• Further trials are needed to investigate if RBC transfusion in acute ICH may lead to improved outcome.

## Abbreviations

ANOVA: analysis of variance; APACHE: acute physiology and chronic health evaluation; CI: confidence interval; CT: computed tomography; HB: hemoglobin; ICH: intracerebral hemorrhage; mRS: modified Rankin score; NIHSS: National Institute of Health Stroke Scale; OR: odds ratio; PaO_2_: partial pressure of arterial oxygen; RBC: red blood cell; SAH: subarachnoid hemorrhage; SD: standard deviation; TBI: traumatic brain injury.

## Competing interests

The authors declare that they have no competing interests.

## Authors' contributions

JD and MS contributed equally and planned and designed the study and performed the statistical analysis. JD wrote the first draft of the manuscript. PH performed data acquisition. MS, KH, MS, LK, JB and SP critically revised the manuscript. TS contributed to conception of the study and critically revised the manuscript.

## Supplementary Material

Additional file 1 **Additional logistic regression models**. Logistic regression models after exclusion of 10 patients who had received red blood cell transfusions. Click here for file
