# Supplementary material for: Low hemoglobin is associated with poor functional outcome after non-traumatic, supratentorial intracerebral hemorrhage
Source: Crit Care. 2010 Apr 14;14(2):R63. doi: 10.1186/cc8961 (PMC2887185; doi:10.1186/cc8961)
Supplement: Additional file 1 — Additional logistic regression models. Logistic regression models after exclusion of 10 patients who had received red blood cell transfusions. [file cc8961-S1.doc]

**Logistic regression models after exclusion of 10 patients who had received RBC transfusions**

**Table 6**: Model to predict unfavourable outcome (mRS 4-6) at discharge (n=186 patients**)**

| **Variable** | **Coefficient** | **P** | **OR (95% CI)** |
| --- | --- | --- | --- |
| NIHSS on admission | 0.259 | <0.001 | 1.30 (1.67-1.45) |
| Intraventricular hemorrhage extension | 1.477 | 0.020 | 4.38 (1.26-15.24) |
| Age | 0.043 | 0.022 | 1.04 (1.01-1.08) |
| Mean HB | -0.242 | 0.074 | 0.79 (0.60-1.02) |

mRS: modified Rankin Scale; OR: odds ratio; CI: confidence interval; NIHSS: National Institutes of Health Stroke Scale;;HB: hemoglobin

**Table 7:** Model to predict unfavourable outcome (mRS 4-6) at 3 months (n=167 patients)

| **Variable** | **Coefficient** | **P** | **OR (95% CI)** |
| --- | --- | --- | --- |
| NIHSS on admission | 0.156 | <0.001 | 1.17 (1.10-1.24) |
| Age | 0.082 | <0.001 | 1.09 (1.04-1.13) |
| Mean HB | -0.286 | 0.017 | 0.75 (0.59-0.95) |

mRS: modified Rankin Scale; OR: odds ratio; CI: confidence interval; NIHSS: National Institutes of Health Stroke Scale; HB: hemoglobin

In summary, after excluding the 10 transfused patients from the multivariate models, mean HB did not remain an independent predictor for unfavourable outcome at discharge but stayed an independent predictor in the model for outcome at 3 months.

The mean HB of the transfused patients was 10.0 mg/dl (6.3-12.0, SD ± 1.6) versus 12.8 mg/dl (7.6-18.4, SD ± 2.0) for patients not receiving transfusions (P<0.001, Mann-Whitney U test). The change of the results in the multivariate model for outcome at discharge therefore may have been caused by the fact that those severe cases were withdrawn from analysis.

However, mean HB remained an independent predictor for outcome at 3 months even after exclusion of the transfused patients - thereby excluding the possibility that RBC transfusion has been the underlying cause for that relationship.
